# Stereospecific lactylation in bacteriology: L/D-lactate partitioning shapes host metabolic-disease axis

**DOI:** 10.3389/fmicb.2025.1693700

**Published:** 2025-10-27

**Authors:** Sirui Wang, Leiliang Zhang

**Affiliations:** ^1^Department of Clinical Laboratory Medicine, The First Affiliated Hospital of Shandong First Medical University and Shandong Provincial Qianfoshan Hospital, Jinan, Shandong, China; ^2^Department of Pathogen Biology, School of Clinical and Basic Medical Sciences, Shandong First Medical University and Shandong Academy of Medical Sciences, Jinan, Shandong, China

**Keywords:** stereoisomer, D-lactate, L-lactate, lactylation, bacteria

## Abstract

Two stereoisomers of lactate, L- and D-lactate, serve as critical conduits for bidirectional communication in host-bacteria interactions and the development of diseases. Lactylation, a novel post-translational modification (PTM), has been linked to the regulation of gene expression, immune responses, and pathogen virulence. This review examines the metabolic pathways of L- and D-lactate, their associated lactylation modifications (K_L-la_, K_D-la_, and K_ce_), and the regulatory mechanisms underlying these processes. We highlight the distinct roles of L- and D-lactate in bacterial metabolism and the implications of lactylation in bacterial infections, exploring their multifaceted impacts on diseases such as infections, metabolic disorders, and neurodegenerative conditions. This review presents novel strategies for targeting stereospecific lactate metabolism and lactylation, and it summarizes key methods for detecting both lactate isomers. Additionally, it provides insights into their clinical applications and outlines future research directions within the context of bacterial-related diseases.

## 1 Introduction

**Figure 1:**
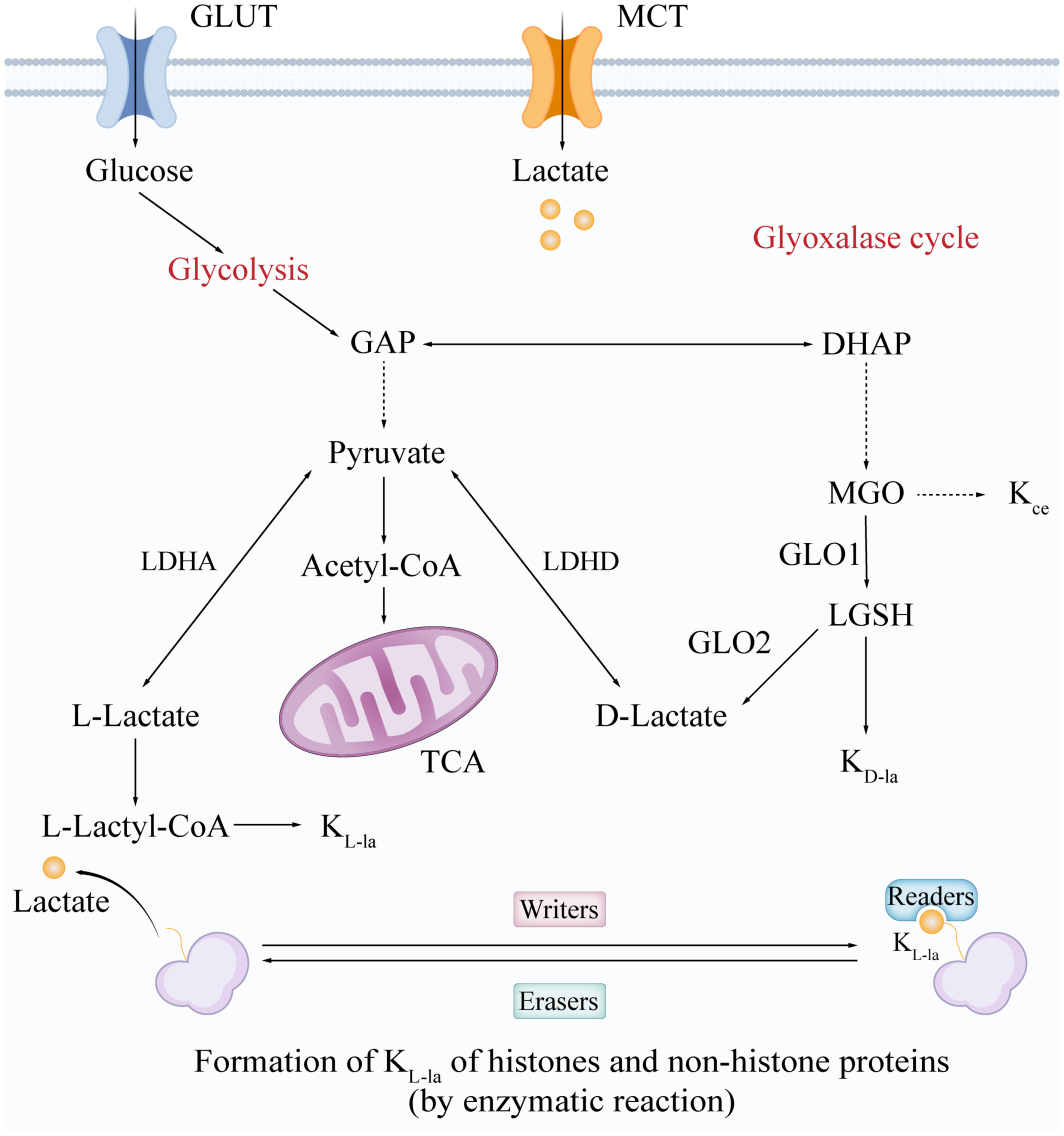
Synthesis and modification mechanisms of L-/D-Lactate and lactylation forms. The formation and structure of lactate and its lactylation isomers are synthesized through two main pathways: glycolysis and the glyoxalase cycle. Glycolysis generates L-lactate and D-lactate via a series of enzymatic reactions. K_L-la_ is formed through an enzymatic reaction regulated by “writers,” “erasers,” and “readers,” while K_D-la_ is produced via an uncatalyzed reaction that involves the generation of LGSH through the glyoxalase cycle. K_ce_ represents one of the MGO adducts. Glucose enters the cell via glucose transporters (GLUT), while lactate enters through monocarboxylate transporters (MCT). GLUT, glucose transporter; MCT, monocarboxylate transporter; GAP, glyceraldehyde-3-phosphate; DHAP, dihydroxyacetone phosphate; LDHA/D, lactate dehydrogenase A/D; acetyl-CoA, acetyl-coenzyme A; TCA, tricarboxylic acid cycle; MGO, methylglyoxal; GLO1/2, glyoxalase I/II; LGSH, S-D-lactoylglutathione; L-Lactyl-CoA, L-lactyl-coenzyme A; K_L-la_, L-lactylation; K_D-la_, D-lactylation; K_ce_, N-*ε*-(carboxyethyl)-lysine.

Lactate, an *α*-hydroxy carboxylic acid, is produced from the reduction of pyruvate as a byproduct of glycolysis. It plays crucial roles as a multifaceted signaling molecule in the regulation of various physiological and pathological processes (Chen J. et al., 2025). Lactate exists in two stereoisomeric forms: L(+)-lactate and D(−)-lactate. L-lactate is predominantly produced by mammalian cells, while D-lactate is preferentially generated by bacterial metabolism (Taylor et al., 2022). This process occurs via lactate dehydrogenase (LDH) during the final step of the glycolytic pathway or through methylglyoxal pathways (Figure 1). A significant difference in biodistribution is observed between these two isomers: L-lactate serves as the dominant form of lactate in humans and eukaryotes, functioning in energy metabolism and signaling, influencing cell survival and gene expression (He et al., 2025). Elevated levels of L-lactate are linked to pathological conditions such as metabolic acidosis and neurodegenerative diseases (Li et al., 2025).

Lactylation is a novel post-translational modification (PTM) of lysine that occurs when a lactate molecule covalently binds to the lysine residues of target proteins (Figure 1) (Zhang et al., 2019). Both histone lactylation (e.g., H3K18la) and non-histone lactylation (e.g., in bacterial toxins) have been found to play key roles in regulating gene expression, immune responses, and pathogen virulence (Tang et al., 2024). Depending on the source of lactate and the mechanism of modification, lactylation can be classified into three main isomers: L-lactylation (K_L-la_), D-lactylation (K_D-la_), and N-*ε*-(carboxyethyl)-lysine (K_ce_) (Zhang D. et al., 2025). These forms exhibit remarkable specificity in their formation mechanisms and biological functions.

K_L-la_ is driven by L-lactate and occurs through an enzymatic reaction mediated by three main classes of enzymes: writers, readers, and erasers. Writers and erasers are the enzymes responsible for adding or removing lactylation modifications, functioning as lactyltransferases and delactylases, respectively (Figure 1). Currently identified writers include three major lysine acyltransferase (KAT) families: p300/CBP (Gao et al., 2025), general control non-depressible 5 (GCN5)-related N-acetyltransferase (GNAT) (Lu et al., 2025), and histone acetyltransferases BAH and MYST, such as KAT8 (Zou et al., 2025), KAT5/TIP60 (Chen et al., 2024), and Oligomerization Domain Containing 1 (HBO1) (Niu et al., 2024). In contrast, prominent erasers include histone deacetylases (HDAC) 1–3 and silent mating type information regulation 2 homologs (SIRT) 1–3 (Moreno-Yruela et al., 2022; Zu et al., 2022; Chen C. et al., 2025; Tsukihara et al., 2025). More recently, SIRT4, another member of the Sirtuin family, has been described as a more effective delactylase (Lv et al., 2025). Notably, studies have also revealed that YiaC functions as a member of the GNAT family, while CobB acts as an eraser in *Escherichia coli* (*E. coli*) (Dong et al., 2022). Additionally, readers are proteins that detect and bind to specific lactylation modifications, thereby influencing gene expression and various cellular processes. Brg1 is the first identified lactonated reader and plays a crucial role in the reprogramming of induced pluripotent stem cells (iPSCs) (Hu et al., 2024). L-lactylation can be directly associated with epigenetically transcriptional reprogramming (Zhao et al., 2025). In contrast, K_D-la_ is generated non-enzymatically from the glyoxalase pathway product lactoylglutathione (LGSH), with its abundance regulated by the activity of thioesterase glyoxalase I/II (GLO1/2) (Figure 1). This suggests that K_D-la_ functions as a safety valve for methylglyoxal (MGO) metabolism (Gaffney et al., 2020). Meanwhile, K_ce_, a byproduct of direct lysine modification by MGO, accumulates abnormally in GLO1-deficient models and modifies histones, albeit at significantly lower levels than K_D-la_. This indicates that K_ce_ may be involved in pathologies associated with oxidative stress (Galligan et al., 2018). Together, these three modifications form a network of lactate metabolism with multilevel interplay involving regulation of epigenetics and enzyme activity, providing a new perspective on how the metabolic microenvironment influences cell fate (Figure 1).

It is evident that lactylation, particularly K_D-la_, exerts a significant influence on bacterial infections. Certain pathogenic bacteria, for instance, have the ability to produce D-lactate via D-lactate dehydrogenase (D-LDH) (Morovic et al., 2024). This lactate isomer, once accumulated within host cells, may modify host proteins through K_D-la_, thereby influencing bacterial gene expression and metabolic pathways, which in turn regulate the production and secretion of virulence factors (Wang et al., 2024; Zhang C. et al., 2025). Moreover, lactylation modifications (K_L-la_ and K_D-la_) have been shown to interfere with host immune responses and cellular metabolism (Sun et al., 2023).

## 2 Species specificity and regulatory factors of bacterial L/D-lactate metabolism

### 2.1 Lactate-producing bacteria and their metabolic regulation

L- and D-lactate, the two stereoisomers of lactate, both play crucial roles in bacterial metabolism. In the bacterial domain, L-lactate primarily arises from the reduction of pyruvate during the final step of anaerobic glycolysis. D-lactate is produced by many bacteria, with certain species, such as *Streptococcus bovis* and *Lactobacilli*, capable of synthesizing substantial amounts through fermentation pathways, while others generate only small quantities via the methylglyoxal pathway. These bacterial products serve as the primary source of D-lactate that mammals subsequently absorb from the gut lumen and their diet (Figure 1) (Jin et al., 2023). The production of both L-lactate and D-lactate is influenced by factors such as ambient pH and the availability of carbon sources. For instance, the activity of LDH in Lactobacillus has been shown to be affected by pH, while the production of D-lactate can be influenced by oxygen levels and nutrient composition (Holland and Pritchard, 1975). Additionally, quorum sensing plays a role in regulating the production of both lactate isomers, allowing bacteria to adapt to environmental changes (Feldman-Salit et al., 2013). These regulatory mechanisms enable bacteria to maintain metabolic flexibility and respond to varying conditions within the host (Li et al., 2022).

### 2.2 The association of L-/D-lactate with diseases

L-lactate has been shown to play a significant role in maintaining intestinal health (Yu et al., 2021). However, dysregulation of its metabolism can disrupt the balance of intestinal flora, leading to various diseases. For instance, increased L-lactate production by intestinal epithelial cells during gut inflammation can enhance the fitness of members of the *Enterobacteriaceae* family, disrupting the microbial balance and exacerbating colitis, along with elevating the risk of inflammation-associated colorectal cancer (Taylor et al., 2022). This lactate imbalance has been demonstrated to impact the equilibrium of intestinal microecology and may trigger a systemic immune response, resulting in chronic inflammation and metabolic disorders (Sinha et al., 2024). Moreover, a link has been established between dysregulated L-lactate metabolism and metabolic diseases, such as obesity and diabetes. This connection arises mainly from the effects of such dysregulation on adipose tissue inflammation and immune responses, which subsequently affects the host’s energy metabolism and insulin sensitivity (Cai et al., 2022; Feng et al., 2022).

Meanwhile, researchers have also linked the accumulation of D-lactate to various diseases. Notably, in short-bowel syndrome, colonic bacterial overgrowth converts unabsorbed carbohydrates into significant amounts of D-lactate, often precipitating D-lactic acidosis (Remund et al., 2023). Patients diagnosed with small intestinal bacterial overgrowth (SIBO) frequently exhibit elevated D-lactate levels, resulting in metabolic acidosis and gastrointestinal symptoms (Rao et al., 2018). From an epidemiological standpoint, diabetic ketoacidosis (DKA) is also significant, as plasma D-lactate concentrations can reach levels of ≥3 mmol L^−1^, contributing to a high anion gap akin to that caused by *β*-hydroxybutyrate (Bo et al., 2013). Furthermore, beyond acute conditions, chronically elevated D-lactate has been implicated in neurodegenerative disorders. In the pathogenesis of Alzheimer’s disease (AD) and Parkinson’s disease (PD), it contributes to neuroinflammation and can result in the formation of advanced glycation end products (AGEs) when its levels rise due to increased glucose metabolism and inadequate detoxification. The accumulation of D-lactate and its precursors, such as MGO, exacerbates mitochondrial dysfunction and neuronal damage, which are hallmark features of these neurodegenerative diseases (de Bari et al., 2019; Dube et al., 2023).

### 2.3 The correlation between bacterial L/D-lactate metabolic processes and lactylation

Three isomeric modifications of lactylation have been identified: K_L-la_, K_D-la_, and K_ce_ (Zhang D. et al., 2025). K_L-la_ is primarily influenced by microenvironmental factors within the host cell, particularly under hypoxic conditions, where the expression level of lactate dehydrogenase A (LDHA) increases (Gao et al., 2025). This elevation promotes the production of K_L-la_. Notably, L-lactate induces histone lactylation and other modifications, which in turn affect gene expression and cellular functions. For example, L-lactate triggers histone lactylation via guanosine triphosphate (GTP)-specific succinyl-CoA synthetase (GTPSCS)-mediated lactyl-CoA synthesis, particularly at the H3K18la site, thereby regulating oncogene expression and enhancing glioma cell proliferation and radioresistance (Liu et al., 2025). Additionally, K_ce_ modification may impact cellular functions under metabolic stress conditions, such as hyperglycemia or glyoxalase 1 (GLO1) deficiency (Galligan et al., 2018). In these scenarios, elevated levels of methylglyoxal result in increased histone adduction and changes in gene transcription.

In contrast, the strong association of D-lactate with bacterial metabolism suggests a potential link to K_D-la_. Research has shown that D-lactate, produced by certain bacteria, can enter host cells through the monocarboxylate transporter protein 1 (MCT1) (Alarcón et al., 2017). LGSH, can undergo K_D-la_ modification; however, its direct involvement in histone lactylation and its impact on disease remain to be fully elucidated. As K_D-la_ has not been detected on histones in wild-type cells (Zhang D. et al., 2025), it may not participate in nuclear processes to the same extent as K_L-la_. Furthermore, additional research is needed to clarify the specific mechanisms and pathways through which D-lactylation may contribute to disease progression.

While the impact of bacterial D-lactate on host signaling has been extensively discussed, the reciprocal influence of how host-derived L-lactate modulates bacterial behavior remains underexplored. Emerging evidence suggests that extracellular L-lactate, particularly under conditions of systemic metabolic stress or within tumor microenvironments, can be taken up by commensal or pathogenic bacteria and incorporated into their proteome via L-lactylation. For instance, *Aggregatibacter actinomycetemcomitans*, an oral pathogen, preferentially utilizes L-lactate over glucose through a dedicated LDH pathway, which is not feedback-inhibited by pyruvate, allowing for sustained L-lactate flux even at high host concentrations (Brown and Whiteley, 2009).

Although direct evidence for L-lactate-driven lactylation of bacterial proteins is still limited, the recent identification of lactyl-CoA-generating enzymes and lactylation sites in *E. coli* and *Salmonella* suggests that host-derived L-lactate could similarly be converted to lactyl-CoA and incorporated into bacterial proteins, thereby modulating gene expression or virulence programs (Wang et al., 2024; Zhang C. et al., 2025). If confirmed, this would complete the reciprocal arm of the L/D-lactate partitioning axis.

## 3 The double-edged sword effect of L/D-lactate in bacterial lactylation and diseases

### 3.1 The role of lactylation in bacterial infection

**Figure 2:**
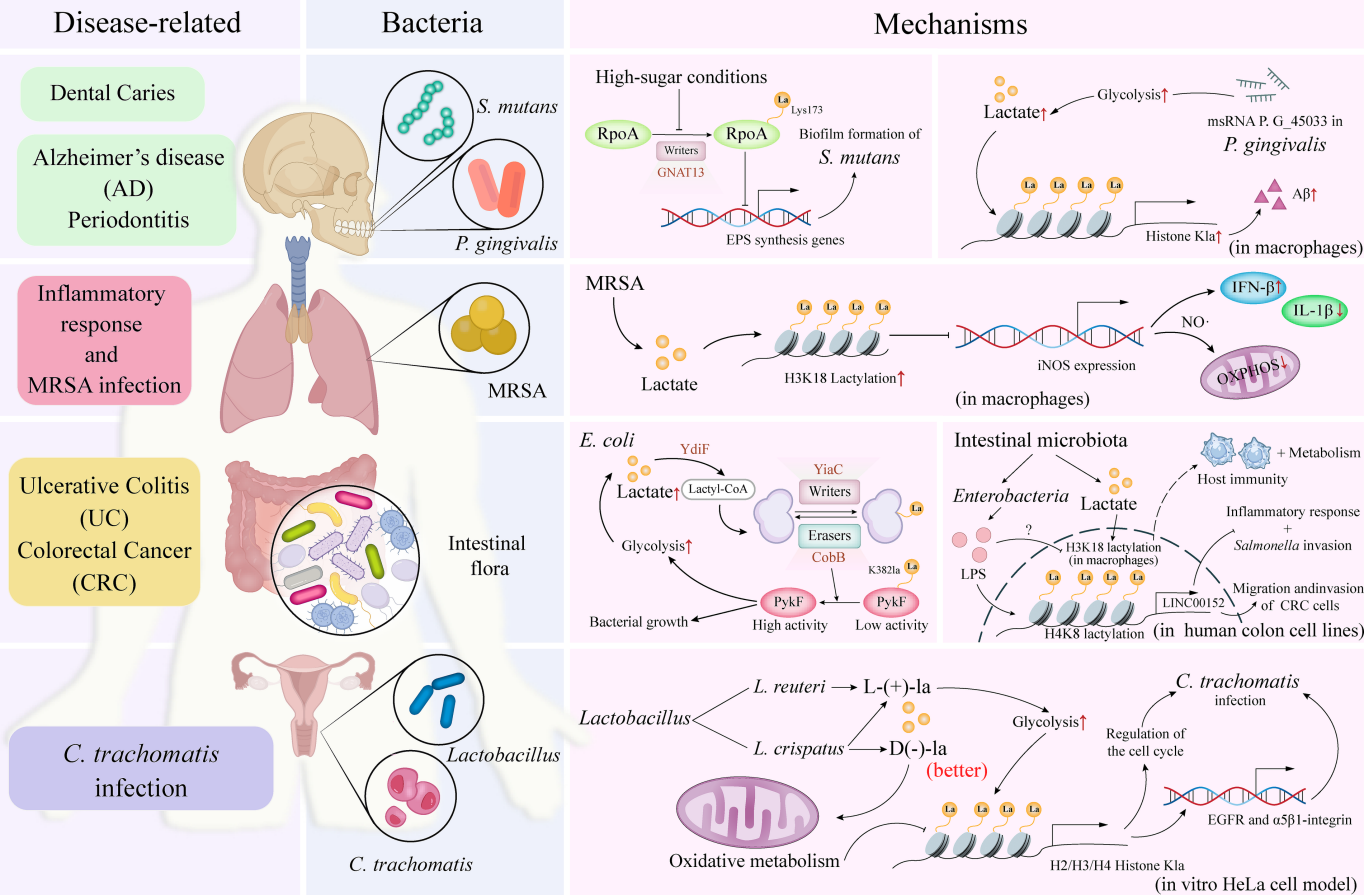
Bacteria-associated lactylation. The distribution of specific bacterial species in various regions of the human body, including the oral cavity, intestines, and vagina, can be correlated with the diseases associated with their infections. By elucidating the specific mechanisms of action of these bacteria in relation to lactylation—a process essential for maintaining microbial ecological balance and promoting overall human health—researchers can deepen their understanding of how these bacteria interact within the human system and impact health outcomes through lactylation. RpoA, RNA polymerase subunit A; GNAT, Gcn5-related N-acetyltransferase; Aβ, amyloid-β; MRSA, methicillin-resistant *Staphylococcus aureus*; iNOS, inducible nitric oxide synthase; IFN, interferon; IL, interleukin; NO, nitric oxide; OXPHOS, oxidative phosphorylation; PykF, pyruvate kinase I; LPS, lipopolysaccharides; lncRNA, long non-coding RNA; EGFR, epidermal growth factor receptor; La, lactate.

Recent studies have highlighted the significant role of lactylation in various biological processes, including bacterial biofilm formation (Li et al., 2023), metabolic regulation (Zhang et al., 2023), transcription and translation (Dong et al., 2022; Wang et al., 2022), pathogen infection, and host interactions (Sun et al., 2021; Reynolds et al., 2024). This underscores the ability of lactylation in bacteria to regulate disease development by engaging in multiple pathways, effectively bridging bacterial–host interactions (Figure 2). It is also important to highlight the roles of YiaC and CobB as acetylases and deacetylases in intestinal bacteria, as established in previous research (Parks and Escalante-Semerena, 2020). Specifically, in *E. coli*, YiaC and CobB function as lactylase and delactylase, respectively, to regulate metabolic processes. YiaC catalyzes lactylation, while CobB modifies metabolic enzyme functions by removing this modification. Notably, CobB regulates the K382la site of PykF through lactate-mediated modifications, thereby promoting glycolysis and supporting bacterial growth (Figure 2) (Dong et al., 2022).

### 3.2 Pathologic mechanisms and the protective effects

The role of lactylation in disease has a dual effect, as it can either promote disease progression or provide a protective role. *Streptococcus mutans* (*S. mutans*), the primary bacterial species associated with dental caries, is predominantly found in biofilms on tooth surfaces (i.e., dental plaque) and has been linked to various diseases and conditions (Mitchell, 2003). Lactylation in *S. mutans* inhibits the synthesis of extracellular polysaccharides—a major component of biofilms (Li et al., 2023). Additionally, the GNAT superfamily enzyme, GNAT13, exhibits lactosyltransferase activity intracellularly, capable of lactosylating the Lys173 residue of RpoA, which subsequently influences biofilm formation (Figure 2).

During *Staphylococcus aureus* (*S. aureus*) infection, lactate levels are elevated, leading to lactylation modifications through the covalent binding of lactate to lysine residues (Wang et al., 2024). Notably, the K84 site of the alpha-toxin is lactylated, and this modification is dependent on the increased lactate concentrations during infection, which enhances the virulence of *S. aureus* and facilitates disease progression.

Furthermore, *Porphyromonas gingivalis* (*P. gingivalis*), a bacterium associated with periodontal disease (Parahitiyawa et al., 2010), expresses the msRNA P. G_45033, which induces the production of amyloid-*β* (Aβ) by enhancing glycolysis and histone lactylation in macrophages (Zhang et al., 2023). Aβ production is significantly linked not only to the development of AD but may also exacerbate the progression of periodontal disease (Figure 2) (Dominy et al., 2019).

Lactylation has also been implicated in the infective capacity of bacterial pathogens. For example, lipopolysaccharides (LPS) from *Enterobacteriaceae* induce the expression of long non-coding RNA (lncRNA) LINC00152 in a human colon cell line, which regulates its promoter region through lactylation. This modification reduces the binding efficiency of the repressor YY1, thus promoting the invasion and migration of colorectal cancer cells. This suggests that gut microbiota can influence host epigenetics through lactylation, thereby participating in disease development (Figure 2) (Wang et al., 2022).

In certain instances, lactylation has been observed to function as a protective mechanism. For example, yeast *Saccharomyces cerevisiae* (*S. cerevisiae*) genetically modified to produce lactic acid directly from glucose instead of ethanol demonstrated anti-inflammatory activity in an ex vivo model and alleviated colitis symptoms by modulating the gut microbiota. In this context, histone H3K18 lactylation may indirectly enhance the production of short-chain fatty acids (SCFAs) by modulating the metabolic and immune responses of host cells, positively affecting the balance of the gut microbiota and overall host health (Sun et al., 2021).

Additionally, a study has revealed that lactylation plays a key role during Methicillin-Resistant *S. aureus* (MRSA) infection by regulating metabolic and inflammatory responses in macrophages. Specifically, MRSA infection triggers metabolic reprogramming in macrophages, which includes disruption of type I interferon (IFN)-mediated oxidative phosphorylation. This process involves the expression and activity of inducible nitric oxide synthase (iNOS) and the subsequent inhibition of *Nos2* gene expression via histone lactylation, thereby limiting nitric oxide (NO) and type I IFN production. Furthermore, chronic exposure to type I IFN inhibits lactate production, which may impact the lactylation process and relate to the resolution of the inflammatory response and host defense mechanisms (Figure 2) (Reynolds et al., 2024).

In investigating the role of D(−)-lactic acid in preventing *Chlamydia trachomatis* (*C. trachomatis*) infection, particularly in an *in vitro* HeLa cell model, researchers have found that the presence of certain Lactobacillus species in the vagina may provide protective effects against *C. trachomatis* infections (Figure 2) (Zalambani et al., 2023). D(−)-lactic acid is believed to be a key antimicrobial compound that plays a central role in host defense. Notably, D(−)-lactic acid not only promotes histone acetylation but also induces lactylation, which may influence gene transcription. The study compared two Lactobacillus species: *Lactobacillus crispatus* (*L. crispatus*) and *Lactobacillus reuteri* (*L. reuteri*). *L. crispatus* produces both D(−) and L(+)-lactic acid isomers, whereas *L. reuteri* produces only the L(+) isomer. The results indicated that *L. crispatus* was more effective in reducing *C. trachomatis* infectivity. Among the findings, D(−)-lactate significantly impacted cell cycle regulation as well as the expression of the epidermal growth factor receptor (EGFR) and α5β1-integrin, suggesting it may prevent *C. trachomatis* infection by altering the metabolic state and gene expression of host cells (Zalambani et al., 2023).

## 4 Detection techniques

The enantiomers of lactic acid (L-lactate and D-lactate) demonstrate significant stereospecificity in bacterial metabolism, signaling regulation, and host–microbe interactions. Clearly distinguishing between these two enantiomers and elucidating their functional differences is essential for understanding bacterial pathogenesis (e.g., regulation of bacterial virulence), clinical diagnosis (e.g., as biomarkers of infection), and industrial applications (e.g., screening for high-purity lactic acid strains) (Bongaerts et al., 1997; Augustiniene and Malys, 2022; Morovic et al., 2024). Table 1 provides a systematic summary of the core methodologies for the simultaneous detection of L- and D-lactate and their associated bacteriological targets. It encompasses a wide range of approaches, including chemical analyses, enzymatic assays, biosensors, and spectroscopic techniques, offering methodological support for studying the stereospecific functional comparisons of lactic acid. However, no single detection method is universally optimal, as each technique has its own trade-offs in terms of cost, complexity, throughput, and compatibility with complex biological matrices. It is important to note that this list is not exhaustive; rather, it highlights a selection of representative references.

**Table 1:** Summary of the main methods for the simultaneous detection of L/D lactate.

Methods	Detection methods	Analytical signal	Characteristics of the methodology	References
HPLC	Chiral column separation	Mass-to-charge ratio	Maturity, cost-efficient, simple operation	Alayande et al. (2020)
LC–MS	High-resolution detection by mass spectrometry	Mass-to-charge ratio	High sensitivity, high selectivity, multi-component analysis	Liu et al. (2024)
UV spectrophotometry	Specific enzymatic reaction (e.g., L-LDH and D-LDH)	Rate of product formation (e.g., NADH concentration), absorbance change rate	Rapid, reagent-minimized, cost-efficient	Zhao et al. (2012)
Enzyme electrode method	Specific enzymatic reactions	Electrode current, oxygen consumption	Rapid, real-time and high-sensitivity detection, suitable for field use	Burstein et al. (1986)
Biosensor based on allosteric transcription factor LldR	Allosteric transcription factor LldR binding, alphaScreen technology	Fluorescence intensity measurement	High sensitivity, high selectivity, suitable for complex samples	Xiao et al. (2022)
Fluorescent biosensor based on DNA aptamers	Specific binding of DNA aptamers to L-lactate	Fluorescence intensity measurement	High specificity, high sensitivity, rapid response, suitable for complex biological samples	Huang and Liu (2023)
NMR spectroscopy	Utilization of chiral shift reagent for D- and L-lactate detection	Chemical shift differences in ^1^H NMR spectra	High sensitivity, high selectivity, suitable for complex samples	Suh et al. (2021)

### 4.1 High-performance liquid chromatography (HPLC) with chiral stationary phases

HPLC with chiral stationary phases is a method that allows for the simultaneous separation of L-lactate and D-lactate in a single analytical run (Ibrahim et al., 2023). This method offers high resolution and sensitivity, with a wide linear range of 0.1–10 mM for both lactate enantiomers, ensuring accurate quantification. It provides excellent baseline resolution, with retention times of approximately 25.5 min for L-lactate and 32.9 min for D-lactate, resulting in a resolution greater than 1.5.

This technique is particularly useful in bacterial fermentation processes, where it can monitor changes in Lactobacillus metabolism under varying pH conditions. The simplicity of operation and the maturity of the technology make it a reliable choice for routine analyses in both research and industrial settings. The method’s characteristics—such as its maturity, cost-efficiency, and ease of operation—make it highly suitable for a wide range of applications, from quality control in the food industry to detailed metabolic studies in microbiology (Alayande et al., 2020). However, the 30-min run time and the high cost of chiral columns limit its application for high-throughput work.

### 4.2 Liquid chromatography-mass spectrometry (LC–MS)

LC–MS has emerged as a powerful analytical tool for the simultaneous detection and quantification of L- and D-lactate in complex biological samples. This technique leverages the high-resolution detection capabilities of mass spectrometry, allowing for the precise identification of enantiomers based on their distinct mass-to-charge ratios. This feature is crucial for achieving high sensitivity and selectivity, which are essential for multi-component analysis in a single run. For instance, LC–MS has been successfully applied to the simultaneous quantification of short-chain fatty acids (SCFAs) and D/L-lactate in ruminal fluid, achieving a limit of detection (LOD) of 0.01 μg/mL while demonstrating excellent accuracy and reproducibility (Gao et al., 2025). Additionally, LC–MS has been utilized to elucidate the specific upregulation of L-lactate in response to hypoxia, highlighting its unique role in cellular adaptation (Liu et al., 2024). These applications underscore the robustness and versatility of LC–MS in metabolic studies, particularly in understanding the roles of lactate in hypoxia and microbiome analysis. However, specialized instruments and labor-intensive sample preparation limit its use to well-equipped laboratories.

### 4.3 UV spectrophotometry

UV spectrophotometry exploits the stereospecific catalysis of NAD^+^-dependent L- and D-LDH isoenzymes, enabling the simultaneous determination of lactate enantiomers by continuously monitoring the rate of NADH formation (Zhao et al., 2012). The differential electrophoretic mobilities of the enzyme-substrate pairs ensure spatially resolved enzymatic turnover, yielding distinct NADH peaks whose integrated absorbances are directly proportional to the respective enantiomer concentrations. Overall, UV spectrophotometry, represented by dual-enzyme EMMA, circumvents the need for chiral chromatographic columns or derivatization reagents, providing a rapid, reagent-minimized, and cost-effective alternative for high-throughput chiral lactate profiling in complex samples. Nevertheless, the presence of matrix dehydrogenases may distort signals, so the use of clean buffers is advisable.

### 4.4 Enzyme electrode method

The enzyme electrode method offers a rapid and sensitive approach for the simultaneous detection and quantification of L- and D-lactate in complex biological samples by leveraging the stereospecificity of dehydrogenase enzymes. In this method, the respiratory chain from *E. coli*, which includes specific dehydrogenases for L- and D-lactate, is immobilized within a gelatin film that is tanned with glutaraldehyde and mounted onto an oxygen probe (Burstein et al., 1986). This setup allows for the selective oxidation of each lactate enantiomer, with the resulting oxygen consumption being directly proportional to the lactate concentration. Selectivity is enhanced through substrate-specific induction of dehydrogenases, differential permeability of intact versus disrupted cells, competitive inhibition by reaction products, and selective thermodenaturation. These features enable the enzyme electrode to perform multi-component analysis in a single run, providing rapid, real-time, and high-sensitivity detection suitable for field use and rapid metabolic profiling in complex matrices such as blood, fermented foods, and ruminal fluid. However, it is worth noting that electrode fouling necessitates periodic recalibration.

### 4.5 Biosensor based on allosteric transcription factor LldR

A biosensor based on the allosteric transcription factor LldR harnesses the lactate-responsive regulator LldR from *Pseudomonas aeruginosa* (*P. aeruginosa*) PAO1 to achieve simultaneous quantification of D- and L-lactate without chromatographic separation (Xiao et al., 2022). In the optimized BLac-6 configuration, His6-tagged LldR and a biotinylated DNA probe containing the LldR-binding site are immobilized on nickel-chelate acceptor and streptavidin donor AlphaScreen beads, respectively. When lactate is absent, LldR bridges the beads, enabling singlet-oxygen-mediated luminescence at 520–620 nm. The addition of either D- or L-lactate induces an allosteric conformational change that dissociates the complex, quenching the signal proportionally to the total lactate concentration. Since LldR recognizes both enantiomers with equal affinity, the assay provides identical calibration curves regardless of the D-/L-lactate ratio, enabling highly sensitive and selective quantification suitable for complex samples. However, as it reports only total lactate, an additional L-specific assay is needed to determine the isomer ratios.

### 4.6 Fluorescent biosensor based on DNA aptamers

Fluorescent aptamer sensors for L- and D-lactate rely on DNA strands that fold stereoselectively around the target, converting binding events into bright fluorescence signals (Huang and Liu, 2023). After 20 SELEX cycles, two high-affinity sequences were isolated that recognize L-lactate with >7-fold preference over D-lactate and negligible response towards pyruvate or 3-hydroxybutyrate. In a strand-displacement format, each aptamer is pre-hybridized to a quencher strand; lactate binding releases the quencher and restores FAM fluorescence proportional to analyte concentration. The assay responds within seconds across a concentration range of 0.55–20 mM, functions in 90% human serum, and can be multiplexed with a spectrally distinct glucose aptamer for dual-color readout. This system requires no enzymes, cofactors, or oxygen, resulting in high specificity, high sensitivity, and rapid turnaround, making it well-suited for continuous monitoring in complex biological samples. However, long-term *in vivo* stability and the cost of DNA modification still await full validation.

### 4.7 NMR spectroscopy

NMR spectroscopy, enhanced by the chiral shift reagent YbDO3A-(L-alanylamide)3, offers a label-free, one-step method for the simultaneous quantification of D- and L-lactate in complex biological matrices (Suh et al., 2021). The method operates stably within the pH range of 6.0–7.4 and requires no chromatographic separation or derivatization. Complementary CEST NMR corroborates this result through distinct saturation peaks at 165 ppm (D) and 154 ppm (L), enabling robust dual-readout quantification. When applied to erythrocytes and non-small cell lung carcinoma (NSCLC) cell lines, the method demonstrates that glucose alone can increase D-lactate production by up to 65% relative to L-lactate in a GLO1-dependent manner. This highlights the method’s high sensitivity and selectivity for analyzing enantiomeric lactate flux in cancer metabolism. However, the requirement for high-field magnets and expensive ytterbium reagents limits its accessibility for routine use.

## 5 Clinical intervention strategies

The emerging field of lactylation research has highlighted the crucial roles of lactate and lactylation in various physiological and pathological processes. This advancement has led to the development of novel intervention strategies for clinical applications. Regulating the activities of enzymes involved in lactate metabolism is an effective strategy for controlling lactate levels and lactylation. In the context of bacterial infections, such as *Listeria monocytogenes* (*L. monocytogenes*) infection, programmed cell death 6 (PDCD6) deficiency enhances antimicrobial activity by promoting LC3-associated phagocytosis, with lactate levels playing a critical role in this process (Sun et al., 2024). LDHA is induced in response to bacterial challenges. When its activity is genetically or pharmacologically inhibited in macrophages, lactate production decreases, leading to impaired LC3-associated phagocytosis (LAP) and compromised bactericidal activity against intracellular bacteria such as *Listeria monocytogenes* and *Salmonella Typhimurium*. This suggests that LDHA could serve as a potential therapeutic target for modulating the immune response.

Additionally, studies in *Salmonella* have demonstrated that lactylation is dynamically regulated by glycolysis and that inhibiting lactate utilization enhances bacterial lactylation (Zhang C. et al., 2025). Clinical trials have also investigated therapies targeting gut dysbiosis, particularly in chronic fatigue syndrome, where the use of antibiotics and probiotics aims to reduce *Streptococcus* overgrowth and D-lactate production. It has been shown that lower *Streptococcus* counts are associated with improvements in sleep and cognitive symptoms (Wallis et al., 2018).

Innovatively, the development of engineered probiotics has introduced new approaches to regulating lactate levels. For example, genetically modified *Lactobacillus acidophilus* (LH@LA), which co-loads lactate oxidase and catalase, effectively reduces tumor lactate levels, improves the immunosuppressive environment, and remodels the tumor microenvironment by inducing the polarization of M2 macrophages to M1 macrophages through the production of D-lactate (Mao et al., 2025).

## 6 Conclusions and prospects

The exploration of lactate isomers and their lactylation modifications in bacteria is increasingly advancing, as evidenced by a growing body of studies. Researchers are identifying lactate isomers and investigating the mechanisms behind their production, revealing their roles in physiological processes such as bacterial energy metabolism, substance transport, and signaling. For instance, recent research on D-LDH (TP0037) in *Treponema pallidum* (*T. pallidum*) has provided valuable insights into the significance of lactate isomers in bacterial physiology and pathology (Deka et al., 2020). In parallel, attention has intensified on the three forms of lactylation (K_L-la_, K_D-la_, and K_ce_) as studies of protein modifications have progressed. K_L-la_ may regulate bacterial metabolic processes by interfering with the active sites of metabolic enzymes or their interactions with other molecules, potentially impacting host metabolic pathways. Conversely, K_D-la_ and K_ce_ could play roles in bacterial stress responses and protein damage repair.

Looking forward, the field urgently needs stereospecific tools, such as antibodies or nanobodies, that can reliably distinguish K_D-la_ from K_ce_. This is essential for accurately mapping the true abundance and pathogenic relevance of these non-enzymatic marks *in vivo*. At the same time, the L/D-lactate ratio itself should be explored as a predictive biomarker. By coupling a rapid chiral LC–MS/MS readout with metagenomic d-ldh profiling and a stable-isotope tracer, it will be possible to construct a “D-lactate burden score” that can be tested in cohorts with inflammatory or metabolic diseases.

The application of interdisciplinary research methods significantly enhances understanding of lactate metabolism and modification mechanisms in bacteria. High-throughput sequencing has been employed to analyze genes associated with lactate metabolism and modification within bacterial genomes, often combined with mass spectrometry and other techniques to identify and quantify lactate and its modified forms. Nevertheless, the study of lactate isomers and lactylation modifications in bacteria is still in its early stages. Further research is needed to clarify the metabolic pathways governing lactate isomer production and conversion and to elucidate the key enzymes and regulatory factors involved. Additionally, the mechanisms underlying the formation of different lactylation forms require refinement, including the identification of modifying enzymes and the specific recognition processes of modifying substrates.

From a technical perspective, enhancing the sensitivity, accuracy, and efficiency of detection methods is paramount. This can be achieved by developing technologies that facilitate real-time, *in situ* detection of lactate isomers and lactylation modifications in live bacteria, as well as simultaneous detection of different isomers and modifications.

In terms of clinical applications, improved typing methods can lead to the development of rapid and precise diagnostic tools for infectious diseases, laying the groundwork for targeted treatments. This enhancement allows for the detection of specific patterns of lactate isomers and lactylation modifications in clinical samples. Furthermore, probiotic products with targeted functions can be developed to prevent and treat conditions such as intestinal infections and inflammatory bowel diseases by leveraging the mechanisms of action of lactate isomers and lactylation modifications. A comprehensive investigation of the interaction mechanisms between bacteria and hosts will elucidate the roles of lactate isomers and lactylation modifications in maintaining intestinal homeostasis and the emergence of diseases, as well as inform strategies for treating related disorders by regulating lactate metabolism in the microbiome.

In summary, research into lactate isomers and lactylation modifications in bacteria holds great promise. Through detailed exploration of their generation mechanisms, action modes, and regulatory networks, significant breakthroughs are anticipated in bacterial physiology, pathology, and clinical medicine. This investigation will also provide essential theoretical foundations and technical support for developing novel antimicrobial strategies and precision medicine approaches. As research progresses and technologies evolve, this field is expected to yield further benefits for human health.
